# Prevalence of initiation of complementary feeding at 6 months of age and associated factors among mothers of children aged 6 to 24 months in Addis Ababa, Ethiopia

**DOI:** 10.1186/s40795-018-0264-5

**Published:** 2018-12-29

**Authors:** Shikur Mohammed, Tewodros Getinet, Samrawit Solomon, Andrew D. Jones

**Affiliations:** 1 Department of Public Health, Saint Paul’s Hospital Millennium Medical College, Addis Ababa, Ethiopia; 2 Department of Nutritional Sciences, School of Public Health, University of Michigan, Ann Arbor, MI USA

**Keywords:** Complementary feeding, Initiation of complementary foods at 6 months of age, Infant and young child feeding, Ethiopia

## Abstract

**Background:**

Inappropriate complementary feeding practices are a major contributor to poor nutritional status of children under 2 years old in Ethiopia. The Ethiopian Ministry of Health recommends that continued breast feeding beyond 6 months should be accompanied by consumption of nutritionally adequate, safe and appropriate complementary foods. The aim of this study was to determine the prevalence of initiation of complementary feeding at 6 months of age and its associated factors among mothers of children aged 6 to 24 months in Addis Ababa, Ethiopia.

**Methods:**

A cross-sectional study was conducted during January and February of 2017 among 600 mothers with children aged 6 to 24 months in Addis Ababa City. The study participants were selected using systematic random sampling technique through a multistage sampling technique. Data were collected using a pre-tested and structured questionnaire by trained data collectors. Data were entered and analyzed using EpiInfo 7 and SPSS version 21, respectively. We used multivariable binary logistic regression to model the associations of selected independent variables with initiation of complementary feeding at 6 months of age.

**Results:**

Approximately 83% of mothers initiated feeding of complementary foods to their child at 6 months of age. This practice was associated with maternal education (primary education v. no education) Adjusted Odds Ratio (AOR) (95%CI): 2.26(1.19, 4.43)), and home delivery of the child (AOR (95%CI): 0.32 (0.12, 0.82)).

**Conclusion:**

Most mothers in the study initiated feeding of complementary foods to their children at 6 months of age. To further improve complementary feeding practices, mothers should be educated on the benefits of introducing complementary feeding at 6 months of age and the consequences of early or late initiation of complementary feeding on child health.

**Electronic supplementary material:**

The online version of this article (10.1186/s40795-018-0264-5) contains supplementary material, which is available to authorized users.

## Background

It is well recognized that the period from birth to 2 years of age is important for the promotion of optimal growth, health and development of children [1, 2]. Infant and young child feeding (IYCF) practices including breast feeding and complementary feeding practices are a primary determinant of the healthy growth and development of children [2]. Exclusive breastfeeding during the first 6 months of a child’s life and continued breast feeding thereafter combined with appropriate complementary feeding can prevent, respectively, 13 and 6% of under-5 deaths each year in low-income and lower-middle-income settings [3]. Even with optimum breast feeding, however, children are at greater risk of under nutrition if they do not receive sufficient quantities of complementary foods after 6 months of age when breast milk alone is no longer sufficient to meet the nutritional requirements of an infant [1]. Poor nutrition may result not only from a lack of high-quality food, but also from inappropriate timing of initiation of complementary foods given to infants and young children [4, 5].

Early introduction of complementary foods may increase infant morbidity and mortality as a result of reduced ingestion of protective factors present in breast milk [6]. Upon introduction of complementary foods, mothers may also produce less breast milk, which may deleteriously affect the infant’s nutrient intake [6, 7]. Complementary foods may also increase infants’ exposure to infectious pathogens if not properly handled and stored [6]. However, initiating complementary feeding too late may also be harmful to children as children beyond the age of 6 months require complementary foods to meet energy and nutrient requirements for healthy growth [7, 8]. In contexts where environmental sanitation is poor, late introduction of complementary foods may reduce exposure to microbial contaminants through food and other vehicles. However, as infants begin to explore their environments around 6 months of age, they will be exposed to different contaminants. Thus, 6 months of age is the appropriate age to introduce complementary foods [9].

The Ethiopia Ministry of Health recommends that infants and children be exclusively breastfed for the first 6 months of life with no additional liquids or foods given, and that continued breast feeding beyond 6 months should be accompanied by consumption of nutritionally adequate, safe and appropriate complementary foods that help to meet the nutritional requirements of children [4]. However, according to data from the 2016 Ethiopian Demographic and Health Survey (EDHS 2016), exclusive breastfeeding during the first 6 months after birth is not widely practiced in Ethiopia—only 51 to 58% of children age under 6 months are exclusively breast fed the day or night preceding the survey [10, 11]. Furthermore, a study conducted in Mekele Town, Northern Ethiopia found that only 63% of mothers initiated complementary feeding at 6 months of age [12]. Therefore, inappropriate complementary feeding may be contributing to the poor nutritional status of children under 2 years of age in Ethiopia [13, 14].

Though few studies have examined complementary feeding practices in Ethiopia [12, 15, 16], to our knowledge, there are no published studies examining the prevalence of initiation of complementary feeding at 6 months of age and associated factors in Addis Ababa, the country’s capital city. Given different cultural practices, access to education and other resources, it is likely that findings from rural Ethiopia cannot be easily extrapolated to an urban population. Given increasing urbanization in Ethiopia as throughout Sub-Saharan Africa [17], it is important to understand both the prevalence and determinants of key IYCF practices, such as initiation of complementary feeding at 6 months of infant age, to appropriately inform policies centered on improving child nutrition and health among distinct populations.

The overall aim of this study was to determine the prevalence of initiation of complementary feeding at 6 months of age and associated factors among mothers of children aged 6 to 24 months in Addis Ababa City, and to provide a basis for an intervention programme to improve initiation of complementary feeding at 6 months of age in particular, and complementary feeding practices in general in this area.

## Methods

### Study context

The study was conducted in public health facilities of Addis Ababa during January and February of 2017. The total population of the city is estimated to be 3.1 million. There are 11 hospitals and 62 health centers serving the population. Primary health coverage of Addis Ababa is approximately 80% [18].

### Study design and sample

A facility-based cross-sectional study design was used. All mothers with children aged 6 to 24 months attending selected public health facilities during the data collection period were eligible for participation in the study. For the first study objective, we used a single population proportion formula based on the proportion of complementary feeding (63%) [12]; marginal error of 5% (d = 0.05); and standard score corresponding to 95% confidence (Zα/_2_ = 1.96) and a design effect of 1.5. Accordingly, the total sample size was determined to be 538. For the second study objective, the sample size was calculated using statCalc of EpiInfo 7 [12] (see Table 1).

**Table 1:** Parameters used to determine the sample size for the second study objective

Variables	CI (%)	Power (%)	Ratio (unexposed: exposed)	% outcome in unexposed grp	Odds ratio	Deff	Sample size
ANC follow up	95	80	1	24	2	1.5	507
Postnatal care	95	80	1	67	2	1.5	564
Birth preparedness	95	80	1	28	2	1.5	471

The final sample size for this study was 600. A multistage sampling technique was used to reach the sampling units from the public health facilities based on their predetermined patient flow rate. In Addis Ababa, there are 10 sub-cities from which 3 sub-cities were selected randomly and 5 public health facilities were included randomly from each selected sub-city. To allocate the study subjects; first, the average numbers of clients who visited the under-five outpatient department daily were estimated by referencing client registration books for 2 weeks prior to data collection. Then, proportional allocation was made based on the possible number of patients that were expected during the study period and systematic random sampling technique was employed to identify study participants. Finally, the sampling interval was determined by dividing the expected patient within the study period by allocated sample size to each facility.

All mothers of children aged 6 to 24 months had equal chance of being included in the study. Mothers who were not permanent residents of Addis Ababa (i.e., they had lived for less than 6 months in Addis Ababa) or who were unable to speak and hear were excluded from the study.

Data were collected using a structured questionnaire administered in-person by trained nurses who worked in the selected health facilities. The questionnaire was pre-tested among a sample of 30 women (representing 5% of the total sample size for the main study) from non-selected health facilities. Following pre-testing, the questionnaire was revised. The questionnaire was initially developed in English, and was subsequently translated to Amharic. We back translated the Amharic questionnaire into English to confirm the consistency of question formulation.

### Measurement of variables

Initiation of complementary feeding at 6 months of age was the outcome variable in analyses. We defined initiation of complementary feeding at 6 months of age as the introduction of complementary foods at 6 months of age [19]. It was assessed by asking mothers an approximate age they have introduced complementary foods to their child. The independent variables that we examined as potential determinants of initiation of complementary feeding at 6 months of age included maternal age, marital status, educational level of mother and husband, occupation of mother, monthly income, religion and ethnicity, ownership of radio/TV, antenatal care **(**ANC) attendance, postnatal care (PNC) attendance, parity, birth spacing, birth preparedness, place of delivery, child age and sex, maternal education about complementary feeding, and maternal self-report of reasons for timing of initiation of complementary foods. These determinants were identified a priori based on evidence from previous studies [1–14, 20].The following reasons for the timing of initiation of complementary foods were assessed: 1) breast milk is not enough to fulfil the energy requirements of the child; 2) belief that child needs additional foods for growth; 3) belief that child’s stomach can easily digest food; 4) belief that breast milk alone is not sufficient for infants’ growth and development; 5) not enough time to breast feed child; 6) to strengthen the child; 7) to reduce maternal workload; 8) to prevent disease; and 9) exclusive breast feeding improves the mother-child relationship and delays pregnancy (Additional file 1). 

### Statistical analysis

EpiInfo7 statistical software was used for data entry and SPSS version 21.0 (2012; SPSS, IBM Corporation, Armonk, NY) was used for data analysis. Descriptive statistics were calculated summarizing the initiation of complementary feeding at 6 months of age and the independent variables described above. In a first stage, simple binary logistic regression models were used to identify variables associated with initiation of complementary foods at 6 months of age. Variables associated with initiation of complementary foods at 6 months of age with *P* ≤ 0.2 in bivariate analyses were entered into multivariable binary logistic regression models. Adjusted odds ratios (AOR) with 95% confidence intervals (CI) were calculated to measure the strength of associations between variables. Associations were considered statistically significant at the *P* < 0.05 level.

### Ethics approval

The research proposal was approved by the ethical review committee of Saint Paul’s Hospital Millennium Medical College. Permission was sought from the respective health facilities. Oral informed consent of participating mothers was sought after the study aim and benefits were explained. The participation was voluntarily and the right not participate fully or partially were respected.

## Results

### Socio demographic characteristics of participants

A total of 600 mothers with children aged 6–24 months were included in the study. A complete (100%) response rate was obtained. More than half of the mothers (59%) were homemaker by occupation, and 36% attended primary school. The mean age of mothers was 27.7 (SD ± 4.6) years with the range being from 18 to 45 years (Table 2).

**Table 2:** Proportion of initiation of complementary feeding (CF) at 6 months of age by maternal socio demographic characteristics

Explanatory variable	Initiation of CF at 6 months of age		N (%)
	Yes (%)	No (%)	
Mothers’ occupation			
Homemaker	296 (84)	56 (16)	352 (59)
Government employee	99 (85)	18 (15)	117 (20)
Trader	54 (79)	14 (21)	68 (11)
Daily laborer	50 (79)	13 (21)	63 (10)
Mothers’ educational level			
No formal education	63 (72)	24 (28)	87 (15)
Primary school	189 (87)	28 (13)	217 (36)
Secondary school	128 (85)	23 (15)	151 (25)
Above secondary school	119 (82)	26 (18)	145 (24)
Ethnicity			
Gurage	176 (85)	31 (15)	180 (30)
Amhara	150 (83)	30 (17)	109 (18)
Oromo	91 (83)	18 (17)	207 (35)
Others^a^	82 (79)	22 (21)	104 (17)
Antenatal care attendance			
Yes	489 (84)	96 (16)	585 (98)
No	10 (67)	5 (33)	15 (2)
Made a plan for reaching the facility during labor			
Yes	433 (84)	80 (16)	513 (85)
No	66 (76)	21 (24)	87 (15)
Place of delivery			
Health facility	487 (84)	91 (16)	578 (96)
Home	12 (54)	10 (46)	22 (4)
Postnatal care visit			
Yes	377 (85)	68 (15)	445 (74)
No	122 (79)	33 (21)	155 (26)
Parity^b^			
One child	241 (86)	41 (14)	282 (47)
≥ two children	258 (81)	60 (19)	318 (53)
Child sex			
Male	283 (84)	54 (16)	337 (56)
Female	216 (82)	47 (18)	263 (44)
Received information about initiation of CF at 6 months of age			
Yes	484 (84)	93 (16)	577 (96)
No	15 (65)	8 (35)	23 (4)

^a^Silte, Tigray,Wolayta, Gamo. ^b^Number of pregnancies reaching viable gestational age

### Complementary feeding practices

Approximately 83% of mothers initiated complementary feeding to their child at about six months of age (Table 3). The major reasons for initiation at 6 months of age were: 1) believing at this age breast milk is not enough to fulfill the energy requirements of the child (52%), and 2) believing that the child needs additional foods for proper growth at this age (41%) (Table 3).

**Table 3:** Complementary feeding practices and attitudes of participating mothers

Variables	Categories	Frequency	Percent (%)
Initiation of complementary foods	At about 6 months of age	499	83.2
	Before or after 6 months of age	101	17
Reasons for initiation of complementary foods at 6 months of age	At this age breast milk is not enough to fulfill the energyrequirements of the child	310	52
	Belief child needs additional foods for growth at this time	248	41
	Belief the stomach of the child can easily digest food	40	7
Reasons for early initiation of complementary foods	Belief that breast milk alone is not enough for infants’ growth and development	66	11
	Not enough time to breast feed	36	6
	To strengthen the child	34	6
	To reduce maternal workload	33	6
Reasons for late initiation of complementary foods	Disease prevention	30	5
	Exclusive breast feeding improves child-mother relationship and delays pregnancy	19	3
Kinds of commonly introduced foods	Soup	92	15
	Cow milk	265	44
	Porridge	182	30
	Water	145	24
	Fruits	110	18
	Others^a^	174	29

^a^Powdered milk, Enjera, Potata, Bread, Egg, Formula feeding, Mekoroni, Serifam

### Factors associated with initiation of complementary feeding (CF) at 6 months of age

Compared to those mothers without formal education, mothers who attended primary school were approximately two times more likely to initiate complementary feeding at 6 months of age [(AOR (95%CI) = 2.26 (1.19, 4.43)] (Table 4).Mothers who delivered their child at home were 68% less likely to initiate complementary feeding at 6 months of age than mothers who delivered the child in a health facility [AOR(95%CI) = 0.32(0.12, 0.82)] (Table 4).

**Table 4:** Multivariable Logistic regression analysis of factors associated with initiation of complementary feeding at 6 months of age among mothers of children aged 6–24 months

Explanatory variable	Crude OR (95%CI)	Adjusted OR (95%CI)
Mothers’ occupation		
Homemaker	1.0	
Government employee	1.01 (0.58,1.85)	–
Trader	0.73 (0.38,1.40)	–
Daily laborer	0.73 (0.37,1.43)	–
Mothers’ educational level		
No formal education	1.0	1.0
Primary school	2.57 (1.39,4.76)^a^	2.26 (1.19,4.43)^b^
Secondary school	2.12 (1.11,4.05)^a^	1.56 (0.78,3.09)
Above secondary school	1.74 (0.93,3.29)	1.22 (0.65,2.51)
Ethnicity		
Gurage	1.0	
Amhara	0.88 (0.51,1.52)	–
Oromo	0.89 (0.47,1.68)	–
Others^a^	0.66 (0.36,1.20)	–
Antenatal care attendance		
Yes	1.0	1.0
No	0.39 (0.13,1.17)^a^	0.89 (0.24,3.34)
Made a plan for reaching the facility during labor		
Yes	1.0	1.0
No	0.58 (0.34,1.00)^a^	0.69 (0.37,1.26)
Place of delivery		
Health facility	1.0	1.0
Home	0.23 (0.01,0.53)^a^	0.32 (0.12,0.82)^b^
Postnatal care attendance		
Yes	1.0	1.0
No	0.67 (0.42,1.06)^a^	0.75 (0.46,1.23)
Parity		
One child	1.37 (0.89,2.11)^a^	1.34 (0.85,2.13)
≥ two child	1.0	1.0
Child sex		
Male	1.0	–
Female	0.88 (0.57,1.35)	–
Received information about start of CF at 6 months of age		
Yes	1.0	1.0
No	0.36 (0.15,0.87)^a^	0.50 (0.19,1.34)

^a^statistically significant at 20% level of significance;^b^statistically significant at 5% level of significance

## Discussion

The findings from this study showed that the overall prevalence of initiation of complementary feeding at 6 months of age was 83% in children aged 6–24 months in Addis Ababa. Similar studies conducted in Mekele Town, Northern Ethiopia [12], the Amhara region of Ethiopia [19], and Wolaita Sodo town of Southern Ethiopia [15] found that approximately 63, 35 and 71% of women initiated complementary feeding at 6 months of age, respectively. The higher prevalence observed in this study may be due to urban location of the study. Compared to rural areas, mothers in Addis Ababa have better access to health care and health information, particularly about the importance of key IYCF practices [6, 7, 14].

We further observed in our study that maternal education and place of delivery were positively associated with initiation of complementary foods at 6 months of age. Mothers who had attended primary school were more likely to initiate complementary feeding at 6 months of age when compared with mothers with no formal education. Findings from previous studies in Mekele Town, Northern Ethiopia and Amhara region, Ethiopia, respectively, indicated that mothers’educational level was significantly associated with mothers’ initiation of complementary foods at 6 months of age [12, 21]. Mothers with formal education may be more likely to read and understand the benefits of initiation of complementary feeding at 6 months of age for their children. In addition, there is strong evidence that maternal education is associated with improved child-care practices, a reduced odds of stunting, and better ability to access and benefit from interventions [22, 23]. The finding that mothers who have delivered at home were less likely to initiate complementary feeding to their child at 6 months of age may be due to access to better (or any) child feeding information within health facilities.

This study also showed that maternal occupation, parity, having antenatal care follow up, and birth preparedness of mothers were not statistically significantly associated with initiation of complementary feeding at 6 months of age. However, the previously mentioned study from Mekele Town, Northern Ethiopia revealed that these factors were associated with mothers’ initiation of complementary feeding at this age [12]. The observed differences may be due to disparities across the two studies with respect to mothers’ income levels and access to health care during and after pregnancy. The urban women in this study may have better employment opportunities that facilitate higher incomes, as well as access to higher quality health facilities where they may be exposed to health education during their pregnancies.

Though this study provides important policy insights relevant for the promotion of optimal child feeding practices, it did have several limitations. Given that the study was cross-sectional in nature, causality cannot be inferred from the observed associations between independent variables and initiation of complementary feeding at 6 months of age. Nonetheless, this study provides suggestive evidence that might inform the design and prioritization of randomized follow-up experiments to examine the impact of educational interventions within and outside health facilities on initiation of complementary feeding at 6 months of age in Ethiopia. There may have also been imperfect recall about complementary feeding practices by the interviewed mothers. For example, women may have forgotten when they first introduced a given food to a child. However, we expect recall bias may have been minimized given that data collectors were specially trained to probe for the precise timing of introduction of foods. This timing was cross-referenced during the interview with the timing of local events and holidays as well as with the vaccination schedule of the child.

## Conclusion

In conclusion, this study suggests the importance of educating mothers on the benefits of introducing complementary feeding at 6 months of age and the consequences of early or late initiation of complementary feeding on child health. Given the apparent importance of institutional deliveries for conveying this information, providing support and incentives to women to deliver in hospital may be especially important. Home visits to mothers by community health extension workers offer another excellent opportunity for counseling women and encouraging them to optimally feed their children. Generally, improving educational opportunities for girls and women may also be a foundational priority for improving health outcomes of both women and children.

## Additional file

Additional file 1: Part 1: Socio-demographic characteristics of the respondents. Part 2: Mothers reproductive and child related characterists. Part 3: complementary feeding practices and attitudes of participating mothers. (DOCX 42 kb)

## Abbreviations

CF: Complementary feeding
IYCF: Infant and young child feeding
