# Supplementary material for: Prevalence of initiation of complementary feeding at 6 months of age and associated factors among mothers of children aged 6 to 24 months in Addis Ababa, Ethiopia
Source: BMC Nutr. 2018 Dec 29;4:54. doi: 10.1186/s40795-018-0264-5 (PMC7050790; doi:10.1186/s40795-018-0264-5)
Supplement: Supplementary file 1 — Part 1: Socio-demographic characteristics of the respondents. Part 2: Mothers reproductive and child related characterists. Part 3: complementary feeding practices and attitudes of participating mothers. (DOCX 42 kb) [file 40795_2018_264_MOESM1_ESM.docx]

## Consent form and Questionnaire

### Oral informed consent

Hello, Good morning/afternoon? My name is____________ and I am from SPHMMC. We are conducting a study on “**timely initiation of complementary feeding**”. You are kindly requested to participate in this study, your selection is random and because of your coming this institution and have child whose age is between 6 to 24 months. Your participation is voluntarily and you have the right not participate fully or partially, and participating in this study has no benefit and harm for you, but the information that you give is very important in improving future child caring or feeding practices. The data that we get from you will not be analyzed individually rather it will be compiled in a group and will be disseminated through different means to use it for decision making. The interview will take about 20-30 minutes. If you agree to participate, I will start my questioning from the very common characteristics.

“May I continue?” If yes, continue interviewing

If no, thank and stop interviewing.

1. **Questionnaire**

**Part 1. Socio-demographic characteristics of study respondents**

| **No** | **Questions** | **Response** | **Skip** |
| --- | --- | --- | --- |
| 101 | How old are you? (age in year) | --------------- (in years) |  |
| 102 | Which religion do you follow? | 1. Muslim 2. Orthodox 3. Protestant 4. Catholic 5. Others(specify)__________ |  |
| 103 | What is your current marital status? | 1. Married 2. single 3. Separated 4. Divorced 5. Others (specify)___________ |  |
| 104 | What is your highest educational level? | 1. No formal education 2. primary school 3. Secondary school 4. Preparatory school 5. Diploma 6. Others (specify)____________________. |  |
| 105 | Husband educational level? | 1. No formal education 2. primary school 3. Secondary school 4. preparatory school 5. diploma 6. Others(specify)____________________. |  |
| 106 | What is your ethnicity? | 1. Amhara  2. Tigraye  3. Orromo  4. Gurage  5. Silte  6. Others (specify)__________ |  |
| 107 | What is your occupation? | 1. Home maker 2. Government employee 3. Trader 4. Daily laborer 5. Others (specify)____________. |  |
| 108 | What is your monthly average income? (in Eth. birr) | 1.________________. |  |
| 109 | Total number of families in the household? | 1.______________. |  |
| 110 | Do you have Radio or Television? | 1. Yes 2. No |  |

***Part 2****.* ***Mothers’ reproductive and child related characteristics.***

| No | Questions | Response | Skip |
| --- | --- | --- | --- |
| 111 | Number of previous delivery (parity). | ________________ |  |
| 112 | What is your Birth interval (if the mothers have more than one child)? | ___________ |  |
| 113 | How many children less than 2 years are there in your household? | ______________. |  |
| 114 | Age of the child (in months) | ___________months |  |
| 115 | Have you attended antenatal clinic when you were pregnant with the last child? | 1. Yes 2. No |  |
| 116 | How many times have you attended ANC in that specific health facility? | ________times. |  |
| 117 | Did you have birth preparedness plan (or made plan to reach the facility during labor)? | 1. Yes 2. No |  |
| 118 | Where did you give birth? | 1. Health facility 2. Home 3. Other(specify)________. |  |
| 119 | Who assisted during delivery? | 1. Midwife 2. Nurse 3. Physician 4. Other (specify)___________. |  |
| 120 | Was the baby single or twin birth? | 1. Single 2. Twin. |  |
| 121 | Did you have postnatal care visit after delivery of the child? | 1. Yes 2. No |  |
| 122 | Sex of the index child | 1. Male 2. female |  |
| 123 | Does the child ever been vaccinated? | 1. Yes 2. No |  |

**Part 3: Complementary feeding practices and attitudes of participating mothers.**

| No | Questions | Response | Skip |
| --- | --- | --- | --- |
| 124 | Do you currently breast feed? | 1. Yes 2. No |  |
| 125 | At what specific age, did you start (initiate) additional foods to your child besides your breast milk? (in months) | ____________months of child age. |  |
| 126 | What do you think are the reasons for initiation of complementary foods at 6 months of child age? (More than one reason is possible) | 1. belief child needs additional food 2. Breast milk is not enough 3. Baby always crying 4. Six month is the correct age to start Complementary feeding 5. Others (specify)__________ |  |
| 127 | What do you think are the reasons for early initiation of complementary foods? (More than one reason is possible) | 1. breast milk alone is not enough for child growth 2. Not enough time to breast feed 3. It trains the infant how to feed for the future. 4. To reduce maternal workload 5. Others (specify)___________ |  |
| 128 | What do you think are the reasons for late initiation of complementary foods? (More than one reason is possible) | 1. It prevents disease 2. delays pregnancy and improve mother child relationship 3. It decrease the infants need for breast feeding 4. Others (specify)_________ |  |
| 129 | What do you think are the common kinds of foods introduce first time for the child? (More than one food is possible) | 1. Soup 2. Cow milk 3. porridge 4. Other (specify)__________ |  |
| 130 | Have you ever heard about when to start complementary feeding? | 1. Yes 2. No |  |
| 131 | If yes, when to start? | At ___________months |  |
| 132 | Have you received information about the start of complementary food at 6 months of child age? | 1. Yes 2. No |  |

**This is the end of the interview, thank you so much for your response and sacrificing your time. We wish you best!**
